# PSYCHOTROPIC MEDICATION USE AND CHANGES DURING THE HOSPITAL STAY

**DOI:** 10.1093/geroni/igad104.0700

**Published:** 2023-12-21

**Authors:** Elizabeth Galik

**Affiliations:** University of Maryland School of Nursing, Baltimore, Maryland, United States

## Abstract

The purpose of this study was to build on prior research and describe the current use of psychotropic medications among the first 365 older hospitalized patients living with dementia participating in a study focused on optimizing physical activity [The Function Focused Care for Acute Care Using the Evidence Integration Triangle Study (FFC-AC-EIT)] and consider the impact of current recommendations for decreasing use of psychotropics. It was hypothesized that controlling for age, gender, race, comorbidities, cognition, and hospital site (exposure or not to the Function Focused Care Intervention), that there would be a decrease in use of psychotropic medications from admission to discharge. Age, gender, race, comorbidities, admitting diagnosis, and medications (antidepressants, antianxiety medications, anticonvulsants, dementia drugs, antipsychotics, sedative-hypnotics, and opioids) were obtained at baseline and medications were obtained as well at discharge. To compare change over time generalized estimating equations were used. The participants were mostly female (63%), White (69%), and 83.06 years (SD=7.90) old. There was no treatment effect. Antidepressant, antianxiety, anticonvulsant, dementia medication, sedative hypnotic and opioid use remained unchanged. Antipsychotic medication use increased significantly from 16% to 21% at discharge. Some changes were made across drug groups such as change from haloperidol to second generation antipsychotics to decrease risk of side effects. A more focused deprescribing intervention is needed to best address psychotropic medication use among individuals living with dementia when hospitalized.

